# Therapeutic Impact of Ascorbic Acid on Oral and Periodontal Tissues: A Systematic Literature Review

**DOI:** 10.3390/medicina60122041

**Published:** 2024-12-11

**Authors:** Gabriele Ruzijevaite, Enrika Acaite, Egle Jagelaviciene

**Affiliations:** 1 Sypsenos Akademija, Naugarduko Str. 41, LT-03227 Vilnius, Lithuania; gabriele.ruzijevaite@gmail.com; 2 Dental First LT, Kosmonautų Str. 55A, LT-68150 Marijampole, Lithuania; 3 Department of Dental and Oral Pathology, Lithuanian University of Health Sciences, Eiveniu Str. 2, LT-50161 Kaunas, Lithuania; egle.jagelaviciene@lsmu.lt

**Keywords:** vitamin C, ascorbic acid, periodontium, periodontal diseases

## Abstract

*Background and Objectives*: Ascorbic acid (AA), a non-metabolized substance in the human body, is acquired from plant-based foods or supplements and is renowned for its antioxidant and anti-inflammatory properties, widely utilized in medicine, particularly in aesthetic practices. In dentistry, exploring adjunctive therapies like AA has gained traction to complement conventional treatments. This systematic literature review aims to assess the effects of ascorbic acid on oral and periodontal health. *Materials and Methods*: Following PRISMA guidelines, a systematic review was conducted across three electronic databases—PubMed, The Cochrane Library, and ScienceDirect. The review focused on randomized controlled trials and uncontrolled clinical trials published in English between 2018 and 2023, examining ascorbic acid’s impact on oral and periodontal tissues. The search, ending 27 September 2023, identified studies meeting inclusion criteria, assessed using The Cochrane and ROBINS-I bias tools. *Results*: Seventeen publications, involving 811 patients, met the selection criteria. In the study groups, seven out of nine studies showed better outcomes in indicators such as bleeding on probing, plaque index, gingival index, clinical attachment level, periodontal pocket depth, and/or gingival recession depth (*p* < 0.05), compared to the control group. Three studies noted reduced VAS scores posttreatment with AA (*p* < 0.05), while two demonstrated accelerated alveolar healing after tooth extraction. Four publications highlighted ascorbic acid’s efficacy in addressing aesthetic concerns. *Conclusions*: Ascorbic acid emerges as a potentially effective adjunctive therapy for managing oral and periodontal diseases and improving gum aesthetics.

## 1. Introduction

Ascorbic acid (AA) is a low molecular weight, water-soluble organic compound with an acidic taste, which is neither synthesized nor stored in the human body [1]. It can only be obtained from plant-based foods and dietary supplements. Small amounts of this vitamin are necessary to maintain optimal body function. The recommended daily intake of AA is 75 mg/day for women and 90 mg/day for men [2]. AA deficiency occurs after consuming less than 10 mg/day for several weeks [2]. In cases of AA deficiency, symptoms of scurvy develop, such as gum swelling and bleeding, delayed wound healing, changes in dentin structure, osteoporosis, pathological tooth mobility, and the loss of dentition. Patients often report fatigue, malaise, lethargy, and anorexia [2].

Ascorbic acid plays an important role in tissue regeneration. When tissue is damaged, biologically active L-ascorbic acid acts as an antioxidant. It neutralizes oxidants produced during the immune response, reduces oxidative stress, and eliminates free radicals and reactive oxygen species. Thanks to its antioxidant properties, it helps prevent further tissue damage and creates favorable conditions for wound healing [3].

Ascorbic acid is essential for connective tissue synthesis, particularly in the production of collagen. Collagen is a crucial component of connective tissue found in bones, cartilage, teeth, ligaments, gums, and blood vessels [4]. It is part of the extracellular matrix, where an initial protective layer of type III collagen is formed, which is later replaced by a fully functional layer of type I collagen [4]. During collagen synthesis, ascorbic acid participates in the hydroxylation reactions of proline and lysine as an activator of the enzyme prolyl hydroxylase in procollagen [1]. AA provides newly synthesized collagen with tensile strength, allowing it to stretch without tearing [4]. In the absence of AA, the structure of collagen loses stability, resulting in the formation of a deficient protein during synthesis.

The antioxidant properties of ascorbic acid can be utilized in aesthetic procedures for corrective purposes. During oxidative reactions, the effects of ultraviolet radiation, environmental pollution, and other factors on melanin synthesis are reduced [4]. In the active site of the enzyme tyrosinase, which participates in melanin production, AA interacts with copper ions, thereby inhibiting tyrosinase activity and reducing melanin formation [4]. Consequently, ascorbic acid is widely used in dermatology for applications and mesotherapy procedures to treat skin pigmentation. In dentistry, the effect of AA on reducing gum pigmentation has been sparsely researched. However, it is recognized that AA is a safe and effective substance used in minimally invasive procedures [4].

Gum changes that occur during scurvy have led to further investigation into the importance of ascorbic acid for periodontal tissue health. It has been found that individuals with lower levels in their blood plasma are more likely to suffer from advanced stages of periodontal disease compared to those with high concentrations of AA in their plasma [3]. Although studies show a correlation between AA deficiency and periodontal diseases, their relationship and development mechanisms remain unclear [2]. The required amount of AA to prevent scurvy-related gingivitis has been established at 10 mg/day [2]. Current studies are still insufficient to reliably and significantly confirm the amount of ascorbic acid needed to maintain oral health [2,3].

There are clinical studies described in scientific databases about various uses of ascorbic acid in dentistry and their effectiveness, but the collected data has not yet been thoroughly systematized and compared. This literature review aims to highlight the significant use of ascorbic acid in periodontology, as its supplementation during periodontal pathology or surgical treatment is rarely encountered in clinical practice. Moreover, it was observed that the significant benefits of antioxidants are either not emphasized or insufficiently highlighted in publications. Analyzing the literature reveals that there are not many studies confirming the benefits of ascorbic acid in dentistry. The studies included in this review cover several methods of using AA in dentistry. Therefore, the aim of this study is to conduct a systematic review of scientific literature, analyzing the effect of ascorbic acid on reducing periodontal inflammation, postoperative wound healing, and gum aesthetics, and to determine the most effective therapy using AA. This systematic review addresses the following question proposed during the planning phase: does the use of AA have a significant therapeutic effect on oral and periodontal tissues? The research question for the systematic review was formulated using the PICO method [5].

## 2. Materials and Methods

### 2.1. Protocol and Registration

This systematic review of scientific literature was approved by the Center of Bioethics of Lithuanian University of Health Sciences (Permission N, 2023-BEC2-244). The PRISMA (Preferred Reporting Item for Systematic Review and Meta-Analyses) protocol was used to plan this systematic review of scientific literature, formulate the study aim, select scientific publications, assess test relevance, and analyze the selected data [6].

### 2.2. Search Strategy and Data Sources

Scientific publications were searched in three electronic databases, PubMed, Cochrane Library, and ScienceDirect, from 21 February 2023 to 27 September 2023 (date of the last article search). Studies of randomized controlled trials and uncontrolled continuous clinical trials were selected and reviewed. The search was conducted using the primary keywords: “ascorbic acid” and “periodontium.” The detailed search filter applied was as follows: (“Vitamin C” OR “Ascorbic Acid” OR “Ascorbate” OR “L-ascorbate”) AND (“Periodontium” OR “Periodontal” OR “Periodontitis” OR “Periodontal Tissue” OR “Gingiva” OR “Gingival” OR “Gum” OR “Gingivitis” OR “Oral Mucosa” OR “Alveolar Bone” OR “Extraction Socket” OR “Wound”). The search was later expanded, and a review of similar sources was conducted. Additional scientific information was manually analyzed on the ResearchGate network.

Before making a final decision, two reviewers evaluated the titles and abstracts of the publications, and the full text of each selected study was thoroughly analyzed. Any disagreements among the reviewers were discussed. The main difference in researchers’ opinions arose regarding the broad scope of ascorbic acid analysis, but it was ultimately agreed that, due to the limited number of studies and available information, the focus should be on analyzing AA impact on oral tissues in general, not just the periodontium.

### 2.3. Eligibility Criteria

Full-text articles in English published between 2018 and 2023 and examining the effects of AA for periodontal health were selected, and a systematic review was conducted. Scientific reviews, pilot studies, in vitro studies or in vivo studies with experimental animals, non-randomized cohort studies, theses, books, and articles not in English or published before 2018 were excluded. The selection criteria for scientific publications are presented in Table 1.

### 2.4. Data Collection

A final search of articles in the PubMed, Cochrane Library, and ScienceDirect databases using the specified keywords and activated filters resulted in the selection of 107 articles. An additional 2 articles were found in other scientific databases. After removing duplicates, 102 publications remained. The publications were then selected based on their titles and abstracts, with 68 publications being excluded. The remaining articles (n = 34) were evaluated through a full-text analysis according to the research methods and materials used, inclusion and exclusion criteria, article accessibility, and other previously mentioned factors. A total of 17 studies were included in the scientific literature review: 14 randomized controlled clinical trials and 3 uncontrolled continuous studies. Data from the selected articles were recorded in a flow chart that specified the main author, year of publication, study types, methodologies used, as well as periodontal diseases, dosage, and the use of AA in different procedures. The effect of AA on inflammatory processes in periodontal tissues was evaluated using the following indicators: bleeding on probing (BOP), plaque index (PI), gingival index (GI), periodontal pocket depth (PPD), and clinical attachment level (CAL). The main variables for postoperative healing were alveolar depth (AD) and width (measured in mesiodistal and buccolingual directions), wound healing, pain intensity (VAS), alveolitis, tissue edema, restricted mouth opening, and gingival recession depth (GRD). Some studies also assessed radiological parameters: radiographic defect bone density (RDBD), and radiographic linear defect depth (RLDD). The following aesthetic changes were analyzed in the studies: Dummett–Gupta oral mucosa pigmentation index (DOPI), gingival pigmentation index (GPI), surface area (PSA), fraction of melanin area (MAF), gingival luminescence (glow) (L), height of the interdental papilla (TPA), and pain intensity (VAS).

**Figure 1:**
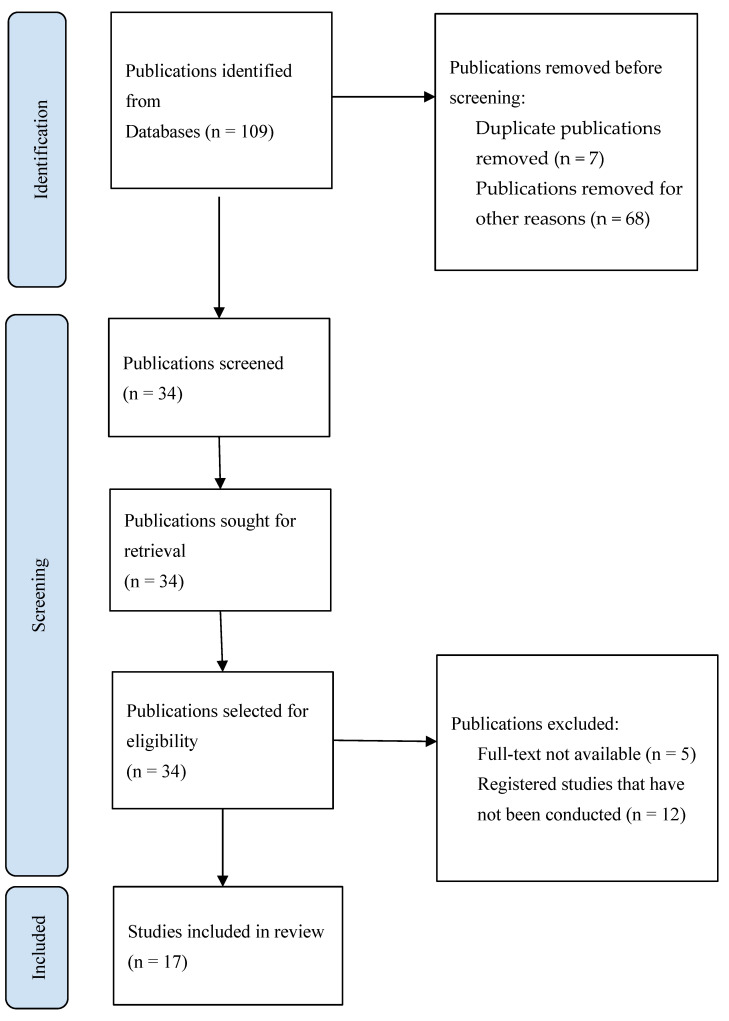
Diagram of PRISMA-based strategy of publication search.

The detailed article selection strategy was prepared and illustrated according to the PRISMA diagram in Figure 1.

The results of the publication search are presented in Table 2.

### 2.5. Risk of Bias Assessment

Randomized clinical trials included in the scientific literature review were analyzed using the Cochrane Collaboration’s tool for assessing the quality of randomized controlled trials [24]. The risk of systematic errors was assessed individually based on six criteria: randomization, allocation concealment, the blinding of participants and researchers, blinded outcome assessment, the completeness of data, and the selective reporting of results. Each criterion was reviewed and evaluated according to an algorithm based on responses to the questions provided. The overall risk of systematic errors in the included studies was rated as low, moderate, or high according to the criteria.

Controlled randomized trials defined as having low [7,8,9,10,12,15,16,17,18,19,20] and moderate [11,13,14] risks of systematic errors, can be considered statistically reliable and included in this literature review. No articles with a high risk of errors were identified during the assessment.

The risk of bias in uncontrolled continuous clinical trials, where the treatment effect is not compared with a control group, was analyzed using the ROBINS-I (risk of bias in non-randomized studies of interventions) assessment tool [25]. Seven criteria were considered: the predictability of treatment effects, selection of participants, classification of interventions, deviations from the intended intervention, incomplete outcome data, outcome assessment, and reporting of selected results. These studies have a low risk [21,22,23], so they can be considered statistically reliable and included in this literature review. No articles with a moderate or high risk of errors were identified during the analysis.

### 2.6. Artificial Intelligence

Artificial intelligence (ChatGPT version 4) was used in writing this article to check the translation into English and to ensure the reference list is correctly formatted in ACS style.

## 3. Results

### 3.1. General Characteristics

The scientific literature review analyzed 17 in vivo studies that described the use of ascorbic acid for the treatment of oral and periodontal tissues and for improving aesthetics. Fourteen publications were randomized controlled clinical trials [7,8,9,10,11,12,13,14,15,16,17,18,19,20], and three were uncontrolled continuous clinical trials [21,22,23]. Among these, there were five single-blind studies [7,10,15,16,18], three double-blind studies [8,9,20], and one triple-blind study [12].

In seven studies [13,14,15,16,18,19,23], the use of AA for inflammation reduction in periodontal tissues was investigated. In six studies [7,8,9,11,20], the effect of the substance on tissue healing after surgical interventions was assessed, while the remaining four studies [10,17,21,22] examined the effect of ascorbic acid on the aesthetic appearance of the gums. Based on these three aspects, the articles were categorized into three groups.

In eight publications [8,11,13,14,15,16,19,20], the participants were diagnosed with chronic periodontitis (PPD > 5 mm, BOP > 10%), while one study [23] diagnosed gingivitis (BOP > 10%, without periodontal attachment loss). One study induced experimental gingivitis [18]. In three studies [7,8,9], the mouth was divided into different segments (split-mouth design), with AA applied as an adjunctive healing agent after tooth extraction. Two studies [9,19] examined the efficacy of preparations containing ascorbic acid. In two studies [13,18], the differences in the effects of natural and synthetic forms of AA were compared.

Different statistical criteria were applied in the scientific articles: ANOVA, Mann–Whitney U, Mann–Whitney Wilcoxon, Chi-square, Bonferroni, Shapiro–Wilk, Friedman, McNemar, Kruskal–Wallis, Student’s *t*-test, Kolmogorov–Smirnov, and Fisher’s exact tests. The efficacy of ascorbic acid preparations was determined based on statistically significant differences in study outcomes between the participant groups, with a significance level set at *p* ≤ 0.05.

A total of 811 participants were involved in the included publications. The sample size varied from five to one hundred twenty-eight participants. The gender distribution among the participants was 40.59% male and 59.41% female. Six authors [13,14,15,21,22,23] did not provide information on gender. The average age ranged from 18 to 45.3 years. Seven authors [13,14,15,17,21,22,23] did not specify the average age of the participants. The studies included participants from two continents—Europe [9] and Asia [7,8,10,11,12,13,14,15,16,17,18,19,20,21,22,23]—and six countries: one study each in Spain [9], China [11], Indonesia [18], and South Korea [19], two studies in Thailand [7,8], three in Egypt [10,13,20], and the majority, eight studies, in India [12,14,15,16,17,21,22,23]. In all cases, the participants were randomly divided into similarly sized groups. Detailed overall study information regarding participants and research characteristics is provided in Table 3.

The inclusion and exclusion criteria of participants across different studies were similar. Excluded were individuals who had taken dietary supplements, antimicrobial or anti-inflammatory medications, or used mouth rinses in the last 2–3 months, or who had undergone periodontal treatment in the last 6 months. Other exclusions included participants with specific dietary needs, those with a history of ascorbic acid allergies, pregnant or breastfeeding women, and those with immunodeficiency diseases, diabetes, or other systemic illnesses.

The analyzed studies compared the natural and synthetic forms of AA, evaluated the effects of AA based on dosage size, assessed the significance of the pharmaceutical form of AA, and examined the total antioxidant capacity (TAOC) of AA in saliva and/or blood serum, as well as its effects when applied through different treatment methods. Two studies [13,18] compared the natural form of AA (guava as a natural source of AA) with its synthetic form, evaluating their significance in gingival inflammatory processes. After the non-surgical treatment of chronic periodontitis, two studies [13,16] administered 500 mg/day AA tablets, while other similar studies [14,15] used 1500 mg/day of ascorbic acid tablets. One study [8] investigated different doses of AA after extraction: 600 mg/day or 1500 mg/day. In Yingcharoenthana et al. study [7], 600 mg/day of AA was assigned to be taken orally and as lozenges following tooth extraction. For mesotherapy with ascorbic acid, different AA injections were explored: Dawar et al. [21] used a concentration of 250 mg/2 mL; El-Mofty et al. [10] used 1 mg/5 mL; and Chaudhary et al. [17] used a 2 mL AA ampoule, containing about 0.1–0.2 mL (200–300 mg of AA). In eight publications [7,8,11,12,13,16,18,19], AA was studied in the form of orally ingested tablets: in seven studies, pure ascorbic acid was used, while in one study [19], AA was one of four active ingredients in the CELC tablet. In five studies [10,17,21,22,23], ascorbic acid was injected, and in three of them [21,22,23], the injections successfully addressed gingival aesthetic issues. In the remaining two studies [10,17], the use of injections was compared with another treatment method: El-Mofty et al. [10] compared mesotherapy with topical therapy (topical gel). Two studies [9,10] used topical vitamin forms, with one of them [9] incorporating ascorbic acid as a component of the gel. In one study [20], pure ascorbic acid was added to platelet-rich fibrin (PRF). Three studies included in the analysis investigated TAOC in saliva [14,15,16], and one of them also assessed it in blood serum [16], collecting saliva and/or serum samples from healthy participants and those with chronic periodontitis. Five studies [13,14,15,16,19] employed non-surgical treatment for chronic periodontal diseases, with four of them administering pure ascorbic acid after treatment: two studies [13,16] used 500 mg/day, while the other two [14,15] used 1500 mg/day. Two studies [12,20] applied surgical treatment for chronic periodontitis. Ascorbic acid was administered post-extraction in three studies [7,8,9]. In their study, Li et al. [11] prescribed 300 mg/day of ascorbic acid tablets after implant placements. Three studies [10,17,21] focused on improving the appearance of the gums.

### 3.2. Characterization of Scientific Studies

#### 3.2.1. Analysis of the Anti-Inflammatory Effects of Ascorbic Acid

Al-Gammal et al. [13] studied 42 patients with chronic periodontitis who did not have any systemic diseases. All participants underwent non-surgical periodontal treatment. The participants were divided into three equal groups, each assigned a daily supplement for 2 months: the first group received 10 mg/day of lycopene (an antioxidant found in fruits and vegetables), the second group received 500 mg/day of AA, and the third group received a placebo. PPD and CAL were measured at the beginning and end of the study using a Williams periodontal probe. It was found that clinical parameters improved in all groups, but no significant difference was observed between them (*p* > 0.05).

Mahajani et al. [14] investigated the effect of AA on patients with chronic periodontitis and its impact on TAOC in saliva. The study included 30 individuals with chronic periodontitis and 30 healthy participants as the control group. All participants were provided with toothpaste that did not contain anti-inflammatory or antioxidant ingredients, instructed in the modified Bass brushing technique, and underwent non-surgical periodontal treatment. The chronic periodontitis group was then divided into two: half were prescribed chewable ascorbic acid tablets (500 mg, 3 times a day) for 60 days, while the other half received no AA supplementation. At the start of the study, saliva samples were collected from all groups, and after 2 months, saliva samples were taken again, but only from the chronic periodontitis groups. TAOC in the saliva was measured using the Koracevic method. At the beginning of the study, the TAOC in the saliva of patients with chronic periodontitis was significantly lower compared to healthy individuals in the control group (*p* < 0.001). After non-surgical periodontal treatment, there was a significant increase in TAOC levels in the saliva, although no significant difference was observed between the two groups of patients with chronic periodontitis (*p* = 0.276). Clinical indicators such as PI, GI, BOP, PPD, and CAL were also evaluated. In patients with chronic periodontitis, clinical measurements were taken before non-surgical treatment, and again after 30 and 60 days. The only significant difference observed between the two chronic periodontitis groups was in the BOP: after 60 days, the group that did not receive AA showed a BOP of 25.17 ± 2.3, while the group that received AA had a BOP of 19.78 ± 1.45 (*p* < 0.001). Clinical periodontal indicators were not assessed for the healthy participants.

Raghavendra et al. [15] conducted a similar study with a larger sample size, involving 50 healthy individuals and 50 patients with chronic periodontitis. The results mirrored those of the previous study [14], showing that supplementing the treatment of chronic periodontitis with AA significantly reduced the BOP (*p* < 0.001). In both studies [14,15], the groups of patients with chronic periodontitis who additionally consumed ascorbic acid experienced an approximately 45% reduction in BOP over the course of two months.

Nisha et al. [16] analyzed the effects of non-surgical periodontal therapy combined with AA supplementation on TAOC in blood serum and saliva in patients with chronic periodontitis. The study included 105 participants, with 70 suffering from chronic periodontitis and 35 healthy individuals serving as the control group for TAOC. The patients with periodontitis were divided into two groups: one received only non-surgical periodontal treatment, while the other group received an additional course of 500 mg/day ascorbic acid for three months after treatment. TAOC samples were collected at the beginning of the study and after three months. Clinical indicators (PI, GI, CAL, PPD, and BOP) were documented at the start, and after 1, 3, 6, and 12 months. It was found that both blood serum and saliva TAOC levels in periodontitis patients were lower than in individuals with healthy periodontium (*p* < 0.05). After non-surgical periodontal treatment, TAOC levels significantly increased in both groups (*p* < 0.05), but no significant difference was observed between the two groups (*p* > 0.05). Similarly to the study by Al-Gammal et al. [13], supplementing with 500 mg/day of ascorbic acid did not produce significant improvements in periodontal health compared to the control group (*p* > 0.05).

Sahithi et al. [23] aimed to investigate the effectiveness of locally administered ascorbic acid injections in treating gingivitis. The study involved 10 patients diagnosed with gingivitis who underwent non-surgical periodontal therapy. After treatment, the participants were divided into two groups: the first group received intraepithelial injections of 150 mg/0.6 mL concentration of ascorbic acid using insulin needles, while the second group received a saline solution (placebo). One week after the procedure, the first group demonstrated significantly improved clinical indicators, with notable enhancements in the PI (*p* = 0.001) and BOP (*p* = 0.008).

Amaliya et al. [18] investigated the relationship between natural and synthetic forms of AA and the development of experimentally induced gingival inflammation. The study included 48 participants who were not suffering from periodontal diseases. Prior to the experiment, all subjects underwent professional oral hygiene for two weeks and were then divided into three groups: the first group was instructed to consume 200 g of guava fruit daily, the second group received a daily supplement of 200 mg of AA in tablet form along with a glass of water, and the control group was given only a glass of water. Following this preparatory phase, the participants were instructed not to clean their lower teeth while continuing normal cleaning of their upper teeth, with individual acrylic guards placed on the lower teeth. This regime continued for two weeks. PI, GI, and BOP parameters were documented at the beginning of the experimental treatment, as well as after 7 and 14 days. After two weeks, the results indicated that both AA groups (natural and synthetic) had significantly lower GI compared to the control group (*p* < 0.001). However, there was no significant change in BOP in either of the treatment groups compared to the control. When assessing PI, the group consuming guava fruit showed significantly lower values at both 1 and 2 weeks (*p* < 0.05), suggesting the potential benefits of natural AA sources in managing gingival inflammation.

Hong and others [19] conducted a study to investigate the impact of a medication called CELC, which contains ascorbic acid as one of its components, on periodontal tissue inflammation in patients with chronic periodontitis. The study involved 100 participants who underwent non-surgical periodontal therapy. After the initial treatment, the subjects were divided into two groups: the control group received placebo for four weeks, followed by CELC for another four weeks, while the experimental group received CELC for a total of eight weeks. PI, GI, PPD, and CAL clinical indices were documented at the beginning of the study, after four weeks, and after eight weeks. The results showed that after four weeks of CELC treatment, there was a significant reduction in the GI (*p* = 0.015). The Generalized Estimating Equations (GEE) model, which accounted for participants’ age, sex, and visit times, also demonstrated a 2.5-fold improvement in the GI in the experimental group compared to the control group (*p* = 0.022). Other clinical indices did not show significant changes. The intensity of pain caused by chronic periodontitis was assessed using the Visual Analog Scale (VAS). In the experimental group, the VAS significantly decreased compared to the control group after 8 weeks (*p* = 0.027).

The study data are summarized and presented in Table 4.

#### 3.2.2. Analysis of the Regenerative Effects of Ascorbic Acid

Elbehwashy et al. [20] enriched PRF with AA to promote regeneration in the treatment of intraosseous defects in Class III chronic periodontitis. The study involved 20 patients, with 10 assigned to the experimental group who underwent regeneration procedures using PRF combined with ascorbic acid. The control group received standard PRF treatment. Clinical CAL, PPD, GRD, BOP, and PI and radiological RDBD and RLDD parameters were documented before treatment and after 3 and 6 months. After 3 and 6 months, the experimental group showed a significant reduction in GRD compared to the control group, with *p* = 0.029 and *p* = 0.010, respectively. The addition of ascorbic acid to PRF resulted in a significant decrease in RLDD in the experimental group after 6 months (*p* = 0.014).

Yingcharoenthana et al. [7] investigated the effects of systemically and locally administered ascorbic acid on tissue healing after tooth extraction. Thirty patients underwent the extraction of premolars from different segments of the mouth. For 14 days, patients took two 100 mg AA tablets three times a day, which were prescribed to be taken orally or slowly dissolved in the mouth for a combined local and systemic effect of the vitamin. The control group did not receive AA. All participating patients were randomly divided into three groups: Group 1 did not receive AA after the first tooth extraction but received oral tablets after the second extraction; Group 2 did not receive AA after the first extraction but received both oral and dissolving tablets (combined effect) after the second extraction; Group 3 received oral tablets after the first extraction and a combined treatment (both oral and dissolving) after the second. At the first visit, a tooth from one side of the mouth was extracted, and one additional treatment or control was applied according to the group. After 21 days, a tooth from the opposite side was extracted, and the scheduled AA treatment was applied. The AD and radiological bone density were measured immediately after tooth extraction, as well as 7 and 21 days later. In the first group, taking AA systemically (orally) for 21 days after tooth extraction resulted in a significant reduction in AD compared to the control area (*p* = 0.018). In the second group, using the combination of local and systemic ascorbic acid, AD significantly decreased during the 7–21-day interval (*p* = 0.028).

Pisalsitsakul et al. [8] examined the significance of AA dosage on tissue healing after tooth extraction. During the study, 42 patients underwent the extraction of 168 premolar teeth. Three experimental groups were formed, each prescribed to take tablets three times a day for 10 days after tooth extraction: participants in the first group received placebo after the first tooth extraction and 600 mg/d AA after the second; participants in the second group received placebo after the first extraction and 1500 mg/d AA after the second; participants in the third group received 600 mg/d AA after the first extraction and 1500 mg/d after the second. The width and depth of the alveolus were measured after the procedure, at 7 and 21 days. For the first three days, VAS was measured three times a day after tooth extraction, which was lower on days 1–3 for those taking 600 mg/d of ascorbic acid (*p* < 0.05). Seven days after the first group supplemented with 600 mg/d of ascorbic acid post-extraction, a reduction in the size of the alveolus was observed in the mesiobuccal direction (*p* = 0.036). The higher dose of 1500 mg/d of ascorbic acid had no effect on wound healing compared to the lower 600 mg/d dose (*p* > 0.05).

Gonzalez-Serrano et al. [9] investigated the effects of a gel containing 2% propolis extract, 0.2% ascorbic acid, and 0.2% vitamin E acetate applications on tissue healing and complication prevention after the removal of lower third molars. Fifteen patients underwent the extraction of two teeth: after the first extraction, they were instructed to apply the gel with AA three times a day for a week. After the second tooth extraction, they received placebo. After 7 days, with additional applications, the participants reported a lower post-extraction pain threshold on VAS (*p* = 0.007).

Li et al. [11] examined different implantation methodologies: conventional implantation in healthy individuals, implantation in patients with chronic periodontitis, implantation with controlled bone regeneration, and implantation with Bio-Oss alloplast. Each type of implantation group had a control and an experimental subgroup, with the latter prescribed 300 mg/d of AA for one week after the procedure. Compared to the control subgroups, the experimental subgroups exhibited better tissue healing after implantation in patients with chronic periodontitis, with implantation with bone regeneration, or alloplast yielding *p* < 0.0001; *p* < 0.0001; and *p* = 0.002. These subgroups showed not only better but also faster postoperative healing (*p* < 0.0001).

Mukherjee et al. [12] analyzed the effects of ascorbic acid on periodontal healing after the surgical treatment of chronic generalized periodontitis. The study included 60 subjects with chronic periodontitis, half of whom were prescribed a two-week course of 500 mg of AA twice daily after the procedure. According to the data obtained, the experimental group showed significantly better wound healing and a lower GI after 7 days (*p* < 0.001), and on days 3 and 7, there was a lower VAS after surgical treatment compared to the control group (*p* < 0.001). In the AA group (experimental), after 1 month, there was a lower GI (*p* < 0.001), and after 3 and 6 months, there were lower PPD values, with *p* = 0.007 and *p* < 0.001, and after 6 months, there was a lower CAL (*p* = 0.001).

The study data are summarized and presented in Table 5.

#### 3.2.3. Analysis of Ascorbic Acid’s Impact on Gum Aesthetics

In the study by Dawar et al. [21], 250 mg/2 mL concentration of AA was injected into hyperpigmentation-affected areas in eight patients through a mesotherapy course of 4–5 injections. Due to COVID-19, only five participants completed the study. The intensity of pigmentation was assessed using the DOPI scale from zero to three: zero—pink tissues, no pigmentation; one—clinically observable, faint pigmentation, light brown; two—moderate clinically observable pigmentation, brown or mixed brown with pink; and three—pronounced clinical pigmentation, dark brown or blue-black. The extent of pigmentation was measured on the GPI scale from zero to three: zero—no pigmented areas; one—brown or black spots; two—brown or black spots without spread; and three—widespread brown or black pigmentation. All participants showed a reduction in DOPI, GPI, and PSA from their baseline after one month, with values of *p* = 0.040, *p* = 0.050, and *p* = 0.040, respectively, while L increased (*p* = 0.040). Although the color of the gums did not change after the first month, the L continued to change for another two months. A histological MAF examination conducted after three months revealed a decrease in the number of melanocytes (*p* = 0.050). Participants rated the treatment positively: 75% of patients reported excellent aesthetics and color changes, and postoperative pain was rated at three points on the VAS.

El-Mofty et al. [10] compared the effectiveness of AA used via application and mesotherapy in removing pigmentation. The study involved 30 patients, who were divided into two groups: the mesotherapy group received injections of 1 mg/5 mL concentration of AA in the hyperpigmented area, while the application therapy group was treated with a gel containing 10% ascorbic acid applied daily for three months. Both groups showed a spread of pink color after 1 and 6 months. The difference in DOPI between the groups was not significant, but the mesotherapy group exhibited significantly faster color changes after one month (*p* = 0.008). After 6 months, MAF decreased in both groups (gel group *p* = 0.005 and injection group *p* = 0.012); no significant differences were observed between the therapeutic groups. Patients in the mesotherapy group rated the aesthetics of the gums better.

Ahuja et al. [22] applied a mesotherapy treatment method, injecting a course of 150 mg of ascorbic acid in six injections around a depressed interdental papilla in 15 patients. Significant changes in papilla height were observed both after 14 days and after 28 days, with an average change of 0.90 ± 0.418 mm from the initial measurement after 42 days (*p* = 0.009).

Chaudhary et al. [17] conducted a mesotherapy treatment course with injections of 200–300 mg of AA, comparing its effects to the surgical removal of hyperpigmentation. The study included 30 participants, divided equally into two groups, undergoing the mentioned procedures. The results showed that after 3 months, there was no significant difference in pigmentation intensity (*p* = 0.754) or the size of the affected area (*p* = 0.932) between both groups. After 3 months following both procedures, repigmentation occurred in about 33% in both groups, but there was no significant difference between the participant groups (*p* = 0.903). After the procedure, 24 h later, participants in the AA group reported lower VAS (*p* = 0.001).

The study data are summarized and presented in Table 6.

## 4. Discussion

Humans lack the active form of the enzyme L-gulonolactone oxidase, which is required for synthesizing ascorbic acid, thus making it essential to obtain AA from plant-based foods and dietary supplements [26]. Ascorbic acid is essential to produce collagen, a structural protein necessary for maintaining the strength and integrity of connective tissues in the body [26]. Moreover, AA acts as an antioxidant in damaged tissues, promoting tissue regeneration [3]. These mechanisms of ascorbic acid action can positively influence tissue regeneration and wound healing in oral tissues following periodontal disease.

This systematic literature review examines various treatment methods, the origin of ascorbic acid, pharmaceutical forms, and dosages. Interpreting the results of the included studies suggests that the proposed hypothesis is correct: ascorbic acid can effectively improve the healing outcomes of oral and periodontal tissues, reduce signs of inflammation, manage postoperative pain, and correct pigmentation areas. For a significant effect based on intervention, it is essential to properly select the source, form, and dosage of AA.

The analysis of experimental gingivitis showed that AA can inhibit the development of gingiva inflammation, regardless of whether the vitamin is natural or synthetic and present in dietary supplements [18]. It is important to note that natural ascorbic acid should be obtained from minimally processed foods, as this substance is highly unstable [2]. The studies included in the analysis compare the significance of the vitamin’s origin [18] and demonstrate that PI (natural) and GI values (natural and synthetic) vary regardless of the vitamin’s origin. Changes in the periodontium (gingiva) with AA consumption are also confirmed by other researchers [27], discussing the effects of AA as an antioxidant. They note that AA consumption decreases the body’s susceptibility to infection, thereby reducing redness, swelling, and bleeding. It also emphasizes the vitamin’s crucial role in periodontal tissues—its ability to stimulate the synthesis of mature collagen since AA is involved in the formation of collagen and extracellular elements. Higher absolute circulating levels of AA may reduce the risk of periodontitis [27]. A deficiency in AA alters the circulatory system and the structure of capillaries, which is related to gingiva bleeding [28,29].

Dividing the daily dose of 1500 mg of AA helps maintain consistent concentration and stable plasma saturation, which influences bleeding regulation. Therefore, when non-surgical gingivitis treatment is supplemented with intraepithelial AA injections, very positive results can be expected [23]: during mesotherapy, the active substance is slowly injected into tissues, creating an effective vitamin concentration in the area of inflamed gums. The injection zone increases the number of fibroblasts and collagen fibers, and forms new capillaries [28]. Thus, the local use of ascorbic acid may be a superior treatment method compared to other pharmaceutical forms of the vitamin when treating inflammatory conditions of periodontal tissues. AA taken orally or in the form of chewable tablets has limiting factors, such as slow and limited absorption.

It has been mentioned that chronic periodontal diseases lead to oxidative stress and disrupt the balance of oxidants and antioxidants. Mahajani et al. [14], Nisha et al. [16], and Raghavendra et al. [15] assessed the total antioxidant levels present in saliva and blood plasma, which decrease in conditions such as chronic periodontitis. Interestingly, the level of antioxidants can significantly recover after a non-surgical tissue treatment intervention, and the additional consumption of 500–1500 mg/d of AA does not have significant relevance. When healthy individuals consume more than 400 mg/d of AA, blood plasma and circulating cells become saturated with ascorbic acid, and the concentration does not increase over time, with excess vitamin being eliminated [30]. Older individuals, smokers, or those with chronic diseases (including periodontal) require higher doses of AA due to the greater need for antioxidants. In this case, the National Institutes of Health recommends a daily intake of 500 mg of AA [30]. Based on the results of the studies reviewed in this literature review [13,16], it can be assumed that a daily dose of 500 mg of ascorbic acid is insignificant in reducing the anti-inflammatory effects in chronic inflammatory periodontal conditions. However, after non-surgical treatment, prescribing a threefold higher daily dose (1500 mg) significantly reduces gingival bleeding—one of the signs of inflammation [14,15]. Laky et al. evaluated multi-nutrient supplementation, including AA, in addition to nonsurgical treatment of periodontitis. The intake of multi-nutrient supplements resulted in a significantly greater reduction in PPD and BOP from baseline to reevaluation compared to placebo [31].

It is worth discussing tooth extraction and tissue healing afterward. This is one of the most common dental procedures. Unfortunately, wounds often heal slowly and with difficulty. Many local and systemic factors influence healing, one of which is inadequate nutrition and vitamin deficiency. As this literature analysis has shown, AA is crucial for healing processes. It has been demonstrated that the concentration of ascorbic acid in blood plasma decreases due to trauma, which also occurs during surgical procedures [32]. It is hypothesized that AA supplements can accelerate postoperative tissue recovery by maintaining normal blood plasma micronutrient levels. Analyzed studies suggest [7,8] that after tooth extraction, administering an additional 600 mg/d of ascorbic acid can lead to greater shrinkage of the socket in both horizontal and vertical directions, as AA can promote the healing of granulation tissue and connective tissue formation within the alveolus due to its involvement in collagen synthesis [4,7]. A stronger regenerative effect occurs when ascorbic acid is administered as a combination of systemic and local application—taken orally and through lozenges—due to direct absorption through the oral mucosa [7,33]. AA reduces the intensity of pain experienced after procedures [7,8,9,12]. Lower pain may indicate a weaker inflammatory process and a shorter course of it. AA has an anti-inflammatory effect, reducing inflammatory markers, such as C-reactive protein levels, which may result in faster healing and less pain [7,8,9,12,34].

Ascorbic acid is used for treating pigmentation since it inhibits tyrosinase, which is directly involved in the synthesis of the melanin precursor dopaquinone [35,36]. Changes in gingival pigmentation due to melanin accumulation are not pathology; treatment is conducted when an individual has concerns about the overall appearance of their smile. Most commonly, hyperpigmentation is treated surgically, where darkened tissues are mechanically removed with a scalpel, laser, or electrocautery. In the study by Sandhu et al., the use of topical AA after the surgical depigmentation of the gingiva resulted in better healing and overall patient comfort. Without AA, the surgical site showed numerous bleeding spots and erythematous gingiva [37]. In the study by Swarna Meenakshi et al., microneedling with ascorbic acid was used to treat gingival pigmentation and showed aesthetically pleasing results compared to conventional surgical depigmentation. The healing index scores were statistically significantly higher in the microneedling with ascorbic acid group [38]. Discovering effective non-surgical methodologies would allow patients to reduce postoperative discomfort, pain, swelling, and the overall risk of complications. This systematic literature analysis has shown that administering ascorbic acid injections via mesotherapy into hyperpigmented areas can yield excellent clinical and histological changes [10,17,21]. The treatment of pigmentation using mesotherapy and application therapy methods has shown [10] that both therapies can improve the aesthetic appearance of the gingiva, but changes in color are observed earlier and more frequently during mesotherapy, leading to greater patient satisfaction. During mesotherapy, the rapid introduction of ascorbic acid into keratinocytes accelerates the reaction between AA and melanocytes due to the high amount of reactive oxygen radicals [39]. According to the data from the reviewed studies, gingival mesotherapy with AA is the most significant method for utilizing ascorbic acid, as the outcome is identical to that achieved through the surgical removal of hyperpigmented areas. Moreover, this method can successfully correct “black triangles” between teeth. This is important not only for aesthetic reasons; unfilled interdental space can lead to phonetic issues and affect the health of periodontal tissues [10,17,21].

This systematic literature review expands knowledge about the additional use of antioxidants in clinical dental practice for the treatment of oral and periodontal tissues and for performing cosmetic gum procedures. It is a minimally invasive, uncomplicated, inexpensive, and easily accessible adjunctive treatment method. The use of AA is contraindicated in cases of allergy, kidney stones, and blood disorders such as thalassemia, hemochromatosis, and sickle cell anemia [2].

Authors should discuss the results and how they can be interpreted from the perspectives of the previous studies and the working hypotheses. The findings and their implications should be discussed in the broadest context possible. Future research directions may also be highlighted.

## 5. Conclusions

In clinical dentistry, the use of ascorbic acid is of significant clinical importance due to its anti-inflammatory properties, antioxidant effects, and its ability to reduce susceptibility to infection. It can limit infection sites, thus reducing secondary periodontal tissue infections. However, the correct dosage and form of ascorbic acid must be chosen based on the required treatment method, as only in this way can the healing process of oral tissues be accelerated following non-surgical and surgical treatments. Due to AA’s effect on collagen synthesis, the formation of new capillaries, and the maintenance of vascular elasticity, clinical parameters change significantly: there is a reduction in gum bleeding upon probing, a decrease in periodontal pocket depth, improved periodontal plaque and gum indices, changes in clinical attachment level, alveolar bone depth, improved radiographic bone tissue parameters, reduced postoperative pain, and faster, better wound healing.

Ascorbic acid also enhances the aesthetics of the gingiva and the overall appearance of the smile. In minimally invasive mesotherapy with AA, excellent treatment results and aesthetic improvements are achieved in areas of mucosal hyperpigmentation and in reducing ‘black triangles’. However, larger-scale and more detailed studies on the use of ascorbic acid in dentistry are still needed.

## Figures and Tables

**Table 1:** The selection criteria for scientific publications.

Inclusion Criteria	Exclusion Criteria
Articles published from 2018 to 2023.	Articles published before 2018.
Articles published in English.	Articles published in languages other than English.
Full articles access available.	Full article access not available.
Randomized controlled trials and uncontrolled continuous clinical trials.	Literature reviews, pilot studies, non-randomized cohort studies, theses, and books.
In vivo studies conducted with humans.	In vitro studies or in vivo studies with experimental animals.
Studies that used AA preparations.	AA was not used in the study.
Reliable treatment outcomes reported.	The reliability of the treatment was not analyzed or reported.

**Table 2:** Publications included in the systematic literature review.

Author, Year	Title	Type of Study
Yingcharoenthana et al., 2021 [7].	A split-mouth randomized clinical trial to evaluate the effect of local and systemic administration of vitamin C on extraction wound healing	RCT
Pisalsitsakul et al., 2022 [8].	Taking 200 mg vitamin C three times per day improved extraction socket wound healing parameters: a randomized clinical trial	RCT
Gonzalez-Serrano et al., 2021 [9].	Application of propolis extract, nanovitamin C and nanovitamin E to prevent alveolar osteitis after impacted lower third molar surgery. A randomized, double-blind, split-mouth, pilot study	RCT
El-Mofty et al., 2021 [10].	Vitamin C mesotherapy versus topical application for gingival hyperpigmentation: a clinical and histopathological study	RCT
Li et al., 2018 [11].	Role of vitamin C in wound healing after dental implant surgery in patients treated with bone grafts and patients with chronic periodontitis	RCT
Mukherjee et al., 2022 [12].	The role of vitamin C in wound healing in periodontal flap surgery in patients with chronic periodontitis: a randomized controlled trial	RCT
Al-gammal et al., 2021 [13].	Clinical periodontal assessment following the adjunctive use of clinical periodontal assessment following the adjunctive use of lycopene and vitamin C in non-surgical therapy of chronic lycopene and vitamin C in non-surgical therapy of chronic periodontitis: a randomized clinical trial	RCT
Mahajani et al., 2021 [14].	Role of vitamin C in patients with chronic periodontitis and its influence on salivary total antioxidant capacity levels	RCT
Raghavendra et al., 2018 [15].	Vitamin C supplementation as an adjunct to nonsurgical therapy in the treatment of chronic periodontitis: a clinical and biochemical study	RCT
Nisha et al., 2023 [16].	Effect of non-surgical periodontal therapy and vitamin C supplementation on total antioxidant capacity in patients with chronic generalised periodontitis—a randomised controlled trial	RCT
Chaudhary et al., 2023 [17].	Physiological gingival melanin hyperpigmentation treatment with injectable vitamin C and scalpel technique: a randomised controlled clinical trial	RCT
Amaliya et al., 2018 [18].	Effect of guava and vitamin C supplementation on experimental gingivitis: A randomized clinical trial	RCT
Hong et al., 2019 [19].	A randomized, double-blind, placebo-controlled multicenter study for evaluating the effects of fixed-dose combinations of vitamin C, vitamin E, lysozyme, and carbazochrome on gingival inflammation in chronic periodontitis patients	RCT
Elbehwashy et al., 2021 [20].	Clinical and radiographic effects of ascorbic acid-augmented platelet-rich fibrin versus platelet rich fibrin alone in intra-osseous defects of stage-III periodontitis patients: a randomized controlled clinical trial	RCT
Dawar et al., 2022 [21].	Oral mesotherapy technique for the treatment of physiologic gingival melanin hyperpigmentation using locally injectable vitamin C: a clinical and histologic case series	CT
Ahuja et al., 2022 [22].	Evaluation of regenerative potential of locally delivered vitamin C along with microneedling in the treatment of deficient interdental papilla: a clinical study	CT
Sahithi et al., 2023 [23].	Evaluation of anti-inflammatory effect of vitamin c using mesotherapy approach in the treatment of persistent gingivitis	CT

RCT—randomized controlled trial, CT—uncontrolled continuous clinical trial.

**Table 3:** General characteristics of the articles.

No.	Main Author, Publication Year	Study Type	Patient Sample (n)	Gender Distribution	Age/Mean (SD)	Control Group Sample	Postoperative Outcome Monitoring
Inhibition of periodontal tissue inflammation							
1	Al-Gammal et al., 2021 [13].	RCT	42	N/I	N/I	14	2 months
2	Mahajani et al., 2021 [14].	RCT	60	N/I	N/I	30	1 month 2 months
3	Raghavendra et al., 2018 [15].	RCT (SB)	100	N/I	N/I	50	1 month 2 months
4	Nisha et al., 2023 [16].	RCT (SB)	105	F54 M51	45 ± 0.88	35	1 month 3 months 6 months 12 months
5	Sahithi et al., 2023 [23].	CT	10	N/I	N/I	5	1 week
6	Amaliya et al., 2018 [18].	RCT (SB)	48	F29 M19	CG 19.6 ± 1.6 TG 20.2 ± 1.3	16	7 days 14 days
7	Hong et al., 2019 [19].	RCT (DB)	93	F60 M33	CG 43.02 ± 14.3 TG 37.83 ± 12.72	45	4 weeks 8 weeks
Postoperative periodontal tissue healing							
8	Elbehwashy et al., 2021 [20].	RCT (DB)	20	F17 M3	CG 28.2 ± 5.63 TG 32 ± 5.27	10	3 months 6 months
9	Yingcharoenthana et al., 2021 [7].	RCT (SB,SM)	30 (60 teeth)	F21 M9	20.07 ± 2.66	SM	7 days 21 day
10	Pisalsitsakul et al., 2022 [8].	RCT (SB,SM)	30 (128 teeth)	F19 M13	18.68 ± 3.95	SM	7 days 21 days
11	Gonzalez-Serrano et.al, 2021 [9].	RCT (SB,SM)	13 (26 teeth)	F10 M3	21.15 ± 2.03	SM	24 h 48 h 72 h 7 days
12	Li et al., 2018 [11].	RCT	128	F68 M60	44.94 ± 2.75	64	3 days 7 days 14 days
13	Mukherjee et al., 2022 [12].	RCT (TB)	60	F32 M28	CG 42.3 ± 6.8 TG 42.0 ± 9.3	30	7 days 14 days 21 days 1 month 3 months 6 months
Gum aesthetics							
14	Dawar et al., 2022 [21].	CT	5	N/I	N/I	-	1 month 3 months
15	El-Mofty et al., 2021 [10].	RCT (SB)	20	F16 M4	27.2 ± 6.8	-	3 months 6 months
16	Ahuja et al., 2022 [22].	CT	15	N/I	N/I	-	14 days 28 days 42 days
17	Chaudhary et al., 2023 [17].	RCT	30	F18 M12	N/I	15	1 week 2 weeks 3 weeks 4 weeks 3 months

RCT—randomized controlled trial, CT—uncontrolled continuous clinical trial, SM—split-mouth study, SB—single-blind study, DB—double-blind study, TB—triple-blind study, N/I—no information, F—female, M—male, CG—control group, TG—treatment group.

**Table 4:** Characteristics of anti-inflammatory effects of AA.

Author, Year	Interventions Applied According to the Study Groups	BOP (%)	GI	PI	PPD (mm)	CAL (mm)	Other Criteria	Results
Al-gammal et al., 2021 [13].	Group I: NST + 10 mg lycopene OD for 2 months	-	-	-	5.3 ± 0.9; after 2 months: 3.5 ± 0.9.	5.2 ± 1.1; after 2 months: 3.4 ± 1.0.	-	The clinical parameters between the groups did not differ significantly (*p* > 0.05).
	Group II: NST + 500 mg vit C OD for 2 months				5.5 ± 1.0; after 2 months: 4.1 ± 0.8.	5.5 ± 1.6; after 2 months: 4.1 ± 1.2.		
	Group III—control: NST + placebo				5.1 ± 1.23; after 2 months: 4 ± 1.11.	5.0 ± 1.54; after 2 months: 3.9 ± 1.14.		
Mahajani et al., 2021 [14].	CG: NST	69.45 ± 4.17; after 30 days: 23.23 ± 1.25; after 60 days: 25.17 ± 2.3.	2.32 ± 0.14; after 30 days: 1.19 ± 0.32; after 60 days: 1.27 ± 0.31.	2.41 ± 0.62; after 30 days: 0.61 ± 0.21; after 60 days: 0.74 ± 0.31.	3.56 ± 0.51; after 30 days: 2.61 ± 1.71; after 60 days: 2.31 ± 0.81.	5.25 ± 0.87; after 30 days: 4.11 ± 0.55; after 60 days: 3.41 ± 0.43.	TAOC: Healthy patients: 781.32 ± 41.13. Chronic periodontitis patients: CG: 541.91 ± 32.41; after 60 days: 661.71 ± 39.62. TG: 514.16 ± 12.98; after 60 days: 639.74 ± 62.15.	The TAOC in the saliva of patients with chronic periodontitis was lower than that of healthy individuals (*p* < 0.05). There was a significant change in BOP after 30 and 60 days of AA supplementation (*p* < 0.001).
	TG: NST + 500 mg chewable vit C TID for 60 days	64.56 ± 1.56; after 30 days: 19.25 ± 1.99 *; after 60 days: 19.78 ± 1.45 *.	2.29 ± 0.27; after 30 days: 1.33 ± 0.27; after 60 days: 1.32 ± 0.23.	2.39 ± 0.26; after 30 days: 0.59 ± 0.26; after 60 days: 0.76 ± 0.26.	3.59 ± 0.34; after 30 days: 2.82 ± 2.55; after 60 days: 2.55 ± 0.43.	5.28 ± 0.24; after 30 days: 4.19 ± 0.12; after 60 days: 3.63 ± 0.22.		
Raghavendra et al., 2018 [15].	CG—NST	68.07 ± 5.48; after 30 days: 22.71 ± 6.16; after 60 days: 24.09 ± 7.17.	2.30 ± 0.14; after 30 days: 1.14 ± 0.51; after 60 days: 1.21 ± 0.48.	2.45 ± 0.12; after 30 days: 0.64 ± 0.16; after 60 days: 0.79 ± 0.25.	3.40 ± 0.91; after 30 days: 2.52 ± 0.82; after 60 days: 2.20 ± 0.41.	5.16 ± 0.55; after 30 days: 4.04 ± 0.68; after 60 days: 3.36 ± 0.91.	TAOC: Healthy patients: 793.97 ± 34.02. Chronic periodontitis patients: CG: 535.64 ± 75.67; after 60 days: 672.24 ± 88.30. TG: 502.78 ± 59.05; after 60 days: 650.30 ± 72.99.	The TAOC in the saliva of patients with chronic periodontitis was lower than that of healthy individuals (*p* < 0.05). There was a significant change in BOP after 30 and 60 days of AA supplementation (*p* < 0.001).
	TG—NST + 500 mg chewable vit C TID for 60 days	63.37 ± 6.80; after 30 days: 17.06 ± 4.18 *; after 60 days: 18.26 ± 4.20 *.	2.26 ± 0.11; after 30 days: 1.30 ± 0.12; after 60 days: 1.30 ± 0.12.	2.43 ± 0.10; after 30 days: 0.63 ± 0.17; after 60 days: 0.78 ± 0.23.	3.52 ± 0.92; after 30 days: 2.72 ± 0.79; after 60 days: 2.48 ± 0.65.	5.16 ± 0.85; after 30 days: 4.08 ± 0.91; after 60 days: 3.52 ± 1.02.		
Nisha et al., 2023 [16].	CG—NST	57 ± 0.53; after 12 months: 27.14 ± 15.66.	2.33 ± 0.13; after 12 months: 1.14 ± 0.12.	2.43 ± 0.11; after 12 months: 0.54 ± 0.13.	3.42 ± 0.56; after 12 months: 2.18 ± 0.42.	5.18 ± 0.32; after 12 months: 3.29 ± 0.34.	TAOC in serum: Healthy patients: 845.05 ± 86.66. Chronic periodontitis patients: CG: 587.78 ± 79.34; after 3 months: 615.22 ± 67.59. TG: 570.08 ± 88.33; after 3 months: 661.91 ± 65.39. TAOC in saliva: Healthy patients: 743.14 ± 45.04. Chronic periodontitis patients: CG: 556.05 ± 28.20; after 3 months: 654.68 ± 36.86. TG: 551.14 ± 31.94; after 3 months: 650.40 ± 47.61.	The TAOC in the saliva and blood serum of patients with chronic periodontitis was lower than in healthy individuals (*p* < 0.05). After treatment, the clinical parameters did not differ significantly between the groups (*p* > 0.05).
	TG—NST + 500 mg vit C OD for 3 months	59 ± 0.55; after 12 months: 28.14 ± 16.63.	2.38 ± 0.16; after 12 months: 1.12 ± 0.18.	2.46 ± 0.13; after 12 months: 0.55 ± 0.12.	3.56 ± 0.62; after 12 months: 2.19 ± 0.44.	5.16 ± 0.41; after 12 months: 3.18 ± 0.23.		
Sahithi et al., 2023 [23].	NST + placebo	2.75 ± 0.54; after 1 week: 2.65 ± 0.63.	-	2.10 ± 0.60; after 1 week: 1.95 ± 0.67.	-	-	-	Additional AA injections significantly reduced BOP (*p* = 0.008) and PI (*p* = 0.001).
	NST + vit C injection	3.25 ± 0.73; after 1 week: 1.60 ± 0.38 *.		2.15 ± 0.42; after 1 week: 0.40 ± 0.14 *.				
Amaliya et al., 2018 [18].	CG: NST + 14 days experimental gingivitis on lower jaw	2.67 ± 3.15; after 7 days: 11.71 ± 11.66; after 14 days: 31.03 ± 21.55.	0.15 ± 0.12; after 7 days: 0.74 ± 0.31; after 14 days: 1.02 ± 0.30.	0.50 ± 0.27; after 7 days: 2.21 ± 0.52; after 14 days: 2.28 ± 0.45.	-	-	-	With poor hygiene practices, additional intake of AA or consumption of guava fruit significantly reduced the GI (*p* < 0.001) and BOP (*p* < 0.001). Consumption of guava fruit also significantly lowered PI (*p* < 0.05).
	+200 g of guava OD (~260 mg vit C) 14 days before and during the experiment	2.68 ± 3.95; after 7 days: 3.26 ± 3.63; after 14 days: 7.46 ± 11.51 *.	0.19 ± 0.13; after 7 days: 0.17 ± 0.12; after 14 days: 0.29 ± 0.33 *.	0.61 ± 0.32; after 7 days: 1.89 ± 0.54 *; after 14 days: 1.91 ± 0.54 *.				
	+200 mg vit C tablet OD 14 days before and during the experiment	4.01 ± 5.99; after 7 days: 6.81 ± 7.70; after 14 days: 7.88 ± 7.20 *.	0.20 ± 0.18; after 7 days: 0.35 ± 0.23; after 14 days: 0.44 ± 0.22 *.	0.42 ± 0.22; after 7 days: 2.07 ± 0.46; after 14 days: 2.03 ± 0.44.				
Hong et al., 2019 [19].	CG—NST + placebo for 4 weeks, then CELC for 4 weeks	-	1.00 ± 0.46; after 4 weeks: 1.01 ± 0.46; after 8 weeks: 0.90 ± 0.50.	1.50 ± 0.68; after 4 weeks: 1.45 ± 0.68; after 8 weeks: 1.48 ± 0.61.	2.49 ± 0.39; after 4 weeks: 2.47 ± 0.37; after 8 weeks: 2.39 ± 0.36.	2.74 ± 0.69; after 4 weeks: 2.75 ± 0.66; after 8 weeks: 2.72 ± 0.71.	-	Supplementing non-surgical periodontal treatment with AA-containing medications significantly reduced GI over 4 weeks (*p* = 0.015). According to the GEE model, TG showed a greater reduction in GI compared to CG (*p* = 0.022).
	TG—NST + CELC (composition: 150 mg vit C, 10 mg vit E, 30 mg lysozyme, 2 mg carbazochrome) OD for 8 weeks	-	1.19 ± 0.51; after 4 weeks: 1.02 ± 0.44 *; after 8 weeks: 0.95 ± 0.49.	1.61 ± 0.67; after 4 weeks: 1.55 ± 0.58; after 8 weeks: 1.42 ± 0.52.	2.63 ± 0.47; after 4 weeks: 2.52 ± 0.49; after 8 weeks: 2.51 ± 0.51.	2.76 ± 0.87; after 4 weeks: 2.69 ± 0.84; after 8 weeks: 2.72 ± 0.88.		

CG—control group, TG—test group, BOP—bleeding on probing, GI—gum index, PI—plaque index, PPD—periodontal pocket depth, CAL—clinical attachment level, TAOC—total antioxidant capacity, NST—non-surgical periodontal treatment, OD—once daily, TID—thrice daily, *—statistically significant difference between the groups.

**Table 5:** Characteristics of regenerative effects of AA.

Author, Year	Procedure	Interventions Applied According to the Study Groups	Criteria	Results
Elbehwashy et al., 2021 [20]	Treatment of intrabony defects in stage III, class C periodontitis (OFD) + (PRF).	CG—OFD + PRF.	CAL, PPD, GRD *, PI, BOP, RDBD, RLDD *, and GRD: TG—1.45 ± 0.76; after 3 months: 0.55 ± 0.69 *; after 6 months: 0.65 ± 0.82 *. CG—0.55 ± 0.55; after 3 months: 0.45 ± 0.64; after 6 months: 0.75 ± 0.68. RLDD: TG—4.69 ± 0.76; after 6 months: 2.40 ± 0.45 *. CG—pr. 3.98 ± 0.43; after 6 months: 2.35 ± 0.42.	When supplementing with AA, comparing between groups, after 3 and 6 months, there was a reduction in GRD (*p* = 0.29; *p* = 0.010); after 6 months, a reduction in RLDD was observed (*p* = 0.014).
		TG—OFD + PRF + vit C (250 μg/mL).		
Yingcharoenthana et al., 2021 [7].	Split-mouth study—extraction of a premolar on one side, followed by extraction on the other side after 21 days.	Group 1: Tooth 1—control. Tooth 2—test. Systemic use—600 mg/day (2 × 100 mg AA, 3 times a day) for 14 days.	Alveolar width in the MD and BL directions, AD *, and radiographic bone density in the alveolus (at the alveolar neck, middle, and apical thirds). AD reduction in group 1 after 21 days: Tooth 1—90.55; Tooth 2—100 *. AD in group 2 comparing the measured values at 7 and 21 days: Tooth 1—61.91; Tooth 2—90 *.	After tooth extraction, systemic use of AA led to a reduction in AG over 21 days, compared to the control group (*p* = 0.018). Systemic and local use of AA significantly reduced AG from day 7 to day 21 after extraction (*p* = 0.028).
		Group 2: Tooth 1—control. Tooth 2—test. Combination of local and systemic use of AA—600 mg per day for 14 days.		
		Group 3: Tooth 1—systemic use of AA—600 mg per day for 14 days. Tooth 2—combination of local and systemic use of AA—600 mg per day for 14 days.		
Pisalsitsakul et al., 2022 [8].	Split-mouth study—extraction of a premolar on one side, followed by extraction on the other side after 21 days.	Group 1: placebo/600 mg AA per day for 10 days	Alveolar width in the MD * and BL directions, AD, and pain (VAS). Reduction of alveolar width in the MD direction in group 1: Placebo subgroup—48.3% AA subgroup—57.3% *	Seven days after tooth extraction, additional intake of 600 mg/day of AA led to alveolus size reduction in the MD direction (*p* = 0.036). Using 600 mg of AA per day for the first three days after extraction resulted in a lower VAS (*p* < 0.05). No significant difference was observed with a higher dose (1500 mg) of AA (*p* > 0.05).
		Group 2: placebo/1500 mg AA per day for 10 days		
		Group 3: 600 mg/1500 mg AA per day for 10 days		
Gonzalez-Serrano et al., 2021 [9].	Split-mouth study—extraction of lower third molar, followed by extraction on the other side after 1 month.	Control side. After extraction—placebo gel three times a day for 7 days.	Alveolitis, swelling, limited mouth opening, wound healing, and pain (VAS).	VAS after tooth extraction and applying a gel with AA locally for 7 days (*p* = 0.007).
		Test side. After extraction—gel (composition: 2% propolis extract, 0.2% ascorbic acid, and 0.2% vitamin E acetate) three times a day for 7 days.		
Li et al., 2018 [11].	Restoration of tooth arch defects through implantation. All groups were divided into two subgroups: half were a control group (CG), and half were a treatment group (TG)—with 300 mg/day of AA prescribed for 7 days.	A (30)—implantation with controlled bone regeneration.	Wound healing (epithelialization at the incision site, presence of granulation tissue, gum color, and response to palpation) and pain (VAS). Wound healing evaluations after 14 days: A: CG—3.93 ± 0.46; TG—4.87 ± 0.35 *. B: CG—4.07 ± 0.26; TG—4.80 ± 0.41 *. C: CG—4.00 ± 0.66; TG—4.76 ± 0.44 *. D: CG—4.67 ± 0.50; TG—4.67 ± 0.49 *.	After implantation, with additional AA administration, improved wound healing was observed after the intervention at 7 and 14 days, when Bio-Oss was used during implantation (*p* < 0.0001); after 14 days, when controlled bone regeneration was performed during implantation (*p* < 0.0001); and after 14 days, when implantation was performed in patients with chronic periodontitis (*p* < 0.002).
		B (30)—implantation with Bio-Oss.		
		C (32)—implantation in patients with chronic periodontitis.		
		D (36)—implantation without bone substitutes in healthy individuals.		
Mukherjee et al., 2022 [12].	Surgical treatment of generalized chronic periodontitis (Kirkland flap).	TG—after treatment, 500 mg of AA is prescribed, two times a day for 14 days.	Wound healing (Chi-square test), pain (VAS), PI, GI, PPD, and CAL. Wound healing evaluations after 7 days: CG: 10%—good; 90%—very good; TG: 100%—excellent *. GI: CG: after 7 days 0.83 ± 0.24; after 1 month 0.30 ± 0.33. TG: after 7 days 0.15 ± 0.35 *; after 1 month 0.03 ± 0.18 *. PPD: CG: after 3 months 2.38 ± 0.59; after 6 months 2.35 ± 0.68. TG: after 3 months 1.80 ± 0.85 *; after 6 months 1.52 ± 0.75 *. CAL after 6 months: CG: 2.38 ± 0.70; TG: 1.68 ± 0.84 *	After surgical treatment of chronic periodontitis, with additional 1000 mg/day of AA: better wound healing after 7 days (*p* < 0.001); lower VAS on days 3 and 7 post-procedure (*p* < 0.001); reduced GI after 1 week and 1 month (*p* < 0.001); decreased PPD at 3 and 6 months (*p* < 0.007); lower CAL after 6 months (*p* < 0.001); and lower PI at different observation points in the treatment group (*p* < 0.005).
		CG.		

BOP—bleeding on probing, PI—plaque index, PPD—periodontal pocket depth, CAL—clinical attachment level, GRD—gingival recession depth, RDBD—radiographic defect bone density, RLDD—radiographic linear defect depth, CG—control group, TG—test group, AD—alveolar depth, MD—mesiodistal, BL—buccolingual, OFD—periodontal open flap debridement, PRF—platelet-rich fibrin, *—statistically significant difference between the groups.

**Table 6:** Characteristics of AA’s impact on gum aesthetics.

Author, Year	Interventions Applied According to the Study Groups	Criteria	Results
Dawar et al., 2022 [21].	Local injections of AA (500 mg) once a week are administered into the hyperpigmented gingiva in the anterior maxillary or mandibular region. The treatment course consists of 4–5 injections.	MAF, DOPI, GPI, L, PSA, pain (0–10), color changes (0–4), and participants’ satisfaction (0–4). MAF: 50–200 (M102); after 3 months: 30–100 (M52). DOPI: 1.25–2.6 (M2.3); after 1 month: 0.8–1.3 (M1.0); after 3 months: 0.5–1.0 (M0.8). GPI: 2–3 (M3); after 1 month: 1–2 (M1); after 3 months: 1–2 (M1). L: 29.6–45.6 (M44.3); after 1 month: 52.0–58.0 (M55.4); after 3 months: 57.0–65.0 (M60.2).	From the beginning to 1 month, there was a statistically significant improvement in DOPI (*p* = 0.04), GPI (*p* = 0.05), L (*p* = 0.04), and PSA (*p* = 0.04). After 3 months, MAF significantly decreased (*p* = 0.05), and L increased (*p* = 0.04). Patients rated aesthetics highly—3/4, postoperative pain was low—3/10, and color changes were excellent—3/4.
El-Mofty et al., 2021 [10].	G1—mesotherapy group, with AA injections (concentration 1 mg/5 mL) administered weekly in the pigmentation area. The treatment course consists of four injections.	MAF, DOPI, pain, participants’ satisfaction, and MAF: G1—0.84–6.11 (M3.01); after 6 months 0.23–3.57 (M0.87) *. G2—0.16–2.87 (M0.3); after 6 months: 0.16–2.81 (M0.25) *. DOPI: G1—2–3; after 1 month 0–2 *; after 3 months 0–2; after 6 months 0–2. G2—1–3; after 1 month 0–2; after 3 months 0–3; after 6 months 1–2.	Over time, G1 showed a significantly faster change in DOPI (*p* = 0.008). In G1, DOPI significantly decreased after 1 month (*p* = 0.008). Patients were more satisfied in G1 (*p* = 0.011). After 6 months, MAF significantly decreased in both groups: G1 (*p* = 0.005) and G2 (*p* = 0.012).
	G2—gel group, with pigmentation areas treated with ascorbic acid gel once a day for 3 months.		
Ahuja et al., 2022 [22].	AA injections of 150 mg are administered once a week into the depressed interdental papilla. The treatment course consists of six injections.	Interdental papilla height (mm): 3.20 ± 0.274; after 14 days: 3.46 ± 0.456 *; after 28 days: 3.76 ± 0.532 *; after 42 days: 4.10 ± 0.652 *.	A significant difference in interdental papilla height was observed between the baseline and day 28 (*p* = 0.020); baseline and day 42 (*p* = 0.009); after day 14 and day 28 (*p* = 0.005); and after day 28 and day 42 (*p* = 0.007). Using AA injections reduces the “black triangles”.
Chaudhary et al., 2023 [17].	G1—AA injections (2 mL, 200–300 mg of AA) are administered once a week for 4 weeks into the anterior maxillary or mandibular pigmented gingiva.	Pain (VAS), itching (0–4), intensity and extent of pigmentation, and repigmentation. Intensity of pigmentation: G1—2.40 ± 0.63; after 1.47 ± 0.64; G2—2.60 ± 0.51; after 0.53 ± 0.52. Extent of pigmentation: G1—114.07 ± 13.24; G2—114.40 ± 8.72. Repigmentation: G1—32.59 ± 5.14; G2—32.87 ± 7.04. VAS: G1—0.73 ± 0.71; G2—3.33 ± 0.82.	There is no significant difference in pigmentation intensity (*p* = 0.754) and area (*p* = 0.932) between the groups before and after the procedures. There is no significant difference in repigmentation (*p* = 0.903) between the groups after 3 months. In the AA group, VAS is significantly lower 24 h after treatment (*p* = 0.001).
	G2—surgical removal of gingival pigmentation.		

MAF—melanin area fraction, DOPI—Dummett–Gupta oral pigmentation index, GPI—gingival pigmentation index, L—gingival luminescence, PSA—pigmented surface area, M—median, *—statistically significant difference between the groups.
